# Novel carbocationic rearrangements of 1-styrylpropargyl alcohols

**DOI:** 10.3762/bjoc.11.114

**Published:** 2015-06-15

**Authors:** Christine Basmadjian, Fan Zhang, Laurent Désaubry

**Affiliations:** 1 Laboratory of Therapeutic Innovation (UMR 7200), University of Strasbourg - CNRS, Faculty of Pharmacy, 67401 Illkirch, France

**Keywords:** carbocationic rearrangement, cyclopentenones, furans, propargyl alcohols

## Abstract

The dehydration and subsequent cyclization reactions of 1-styrylpropargyl alcohols was examined. In the course of these studies, numerous scaffolds were synthesized, including a furan, a cyclopentenone, an acyclic enone and even a naphthalenone. The diversity of these structural motifs lies in novel cascades of reactions originating from a common carbocationic manifold.

## Introduction

**Scheme 1:**
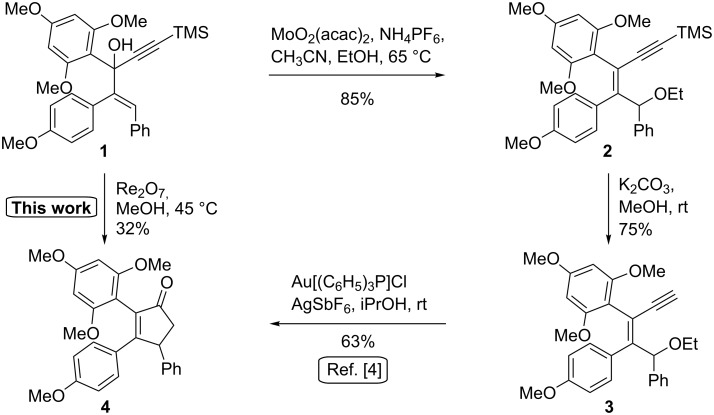
Described synthesis of cyclopentenone **4** using a combination of Mo(VI) and Au(I)-catalyzed reactions and serendipitous discovery of a direct conversion of alcohol **1** into **4** [4].

In the course of our medicinal program on a new class of anticancer agents [1–3], we developed a novel synthesis of cyclopentenones substituted by three different aryl groups (Scheme 1) [4]. This approach combines a molybdenum(VI)-catalyzed etherification of allylic alcohol with a gold(I)-catalyzed intramolecular cyclization process [5–6].

**Scheme 2:**
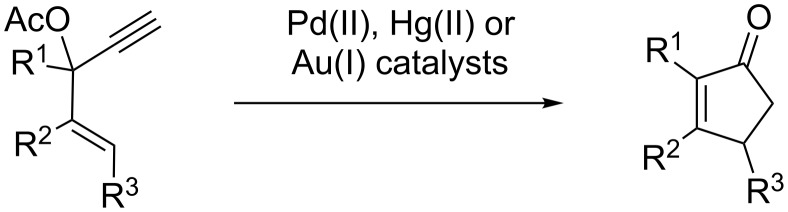
The Rautenstrauch rearrangement.

During the optimization process of this synthesis, we examined several catalysts to transform allylic alcohol **1** into ether **2**, including Re_2_O_7_, which is described to efficiently catalyze this type of transformation [7]. Unexpectedly, instead of obtaining ether **2**, we observed the formation of cyclopentenone **4** in 32% yield. We noticed that this reaction only occurs when Re_2_O_7_ is heated at 45 °C for 15 minutes in MeOH prior to the addition of the substrate. As far as we know, this type of reaction has not been described before. It provides a useful alternative to the Rautenstrauch rearrangement, the main limitation of which lies on the necessity to have the alcohol esterified (Scheme 2) [8]. Indeed, in many cases, this esterification occurs in low yield, or may even be impossible to achieve [4,8–9].

**Scheme 3:**
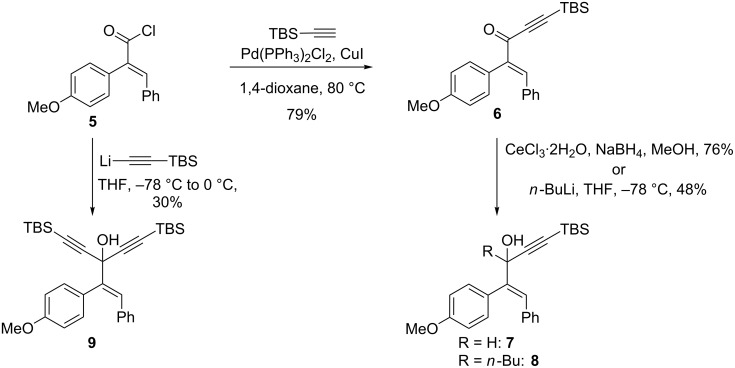
Synthesis of 1-styrylpropargyl alcohols.

The importance of cyclopentenones as intermediates to the synthesis of bioactive compounds prompted us to explore the synthetic potential of this novel rearrangement of 1-styrylpropargyl alcohols (Table 1). Toward this purpose, a number of other substrates were synthesized from the readily prepared acyl chloride **5** and ketone **6** [4] (Scheme 3).

## Results and Discussion

We began our study by applying the reaction conditions developed for alcohol **1** using Re_2_O_7_ (1.5%), MeOH (8 equiv) at 45 °C in DCE (entry 1, Table 1). Gratifyingly, replacing the TMS group by a TBS group improved the yield to 44% (which is superior to the overall yield of the previous three steps synthesis). Along with cyclopentenone **4**, we observed the formation of furan **13** (27% isolated yield).

**Table 1:** Rearrangement of 1-styrylpropargyl alcohols **7**–**10**, **15**, and **16**.

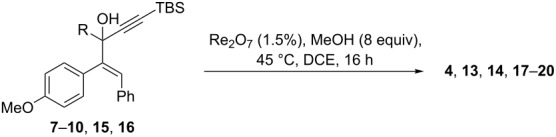			

Entry	R	Isolated products^a^	Yield (%)^b^

1	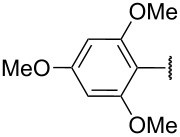 **10**^c^	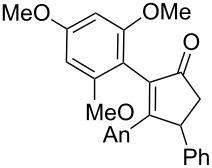 **4**	44
		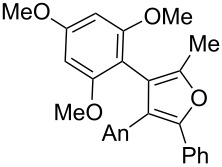 **13**	27
2	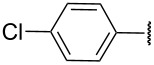 **15**^c^	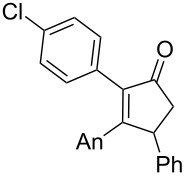 **14**	20
3	Ph **16**^c^	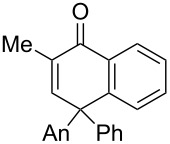 **17**	35
4	H **7**	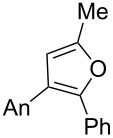 **18**	54
5	*n*-Bu **8**	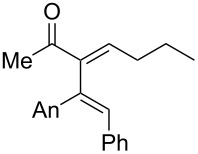 **19**	47
6	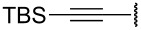 **9**	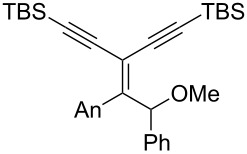 **20**	31

^a^Re_2_O_7_ (1.5%), MeOH (8 equiv), 45 °C, DCE, 16 h. ^b^Isolated yields. ^c^Synthesis described in ref [4]. An = *p*-anisyl.

**Scheme 4:**
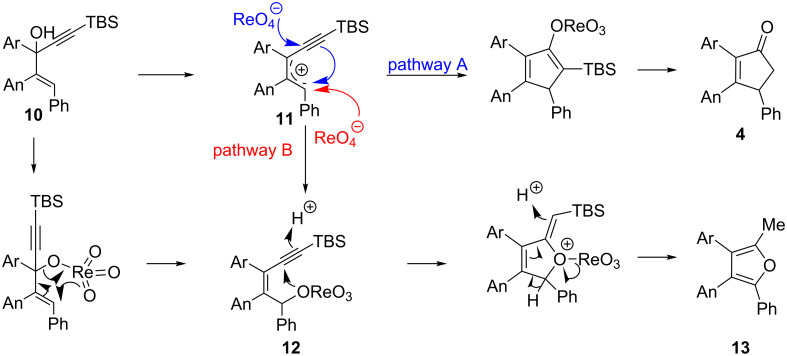
Postulated mechanism for the formation of cyclopentenone **4** and furan **13** (entry 1, Table 1; An= *p*-anisyl).

The proposed mechanism for this reaction is shown in Scheme 4. Both **4** and **13** are expected to arise through the formation of the stabilized carbocation **11** that may evolve through two pathways. This intermediate may either undergo a ring closure due to the nucleophilic character of the silylated alkyne (pathway A) or react with an oxygenated nucleophile, such as the perrhenate anion, to generate the transient intermediate **12** that undergoes an oxo-cyclization to afford an oxonium en route to furan **13** (pathway B). Alternatively, intermediate **12** may result from the allylic [1,3]-transposition of a perrhenate ester [10].

Replacement of the highly electron-donating 2,4,6-trimethoxyphenyl group by a 4-chlorophenyl substituent reduced the yield of cyclopentenone formation to 20% (entry 2, Table 1). No furan or other compound could be isolated. As a result, we turned to the examination of substrate **16** [4], whose unsubstituted phenyl ring is even less amenable to stabilize a carbocationic intermediate. Unsurprisingly, we could not detect any cyclopentenone. Instead, we were amazed to isolate the rearranged ketone **17** (entry 3, Table 1). Indeed, 4,4-diarylnaphthalen-1-ones are highly unusual compounds, whose synthesis is rarely described in literature [11]. The structure of **17** was confirmed by NOE and key HMBC correlations (Figure S1, Supporting Information File 1). Although highly speculative and without any experimental support, a putative mechanism for this unprecedented reaction is proposed in Supporting Information File 1 (Scheme S1).

**Scheme 5:**
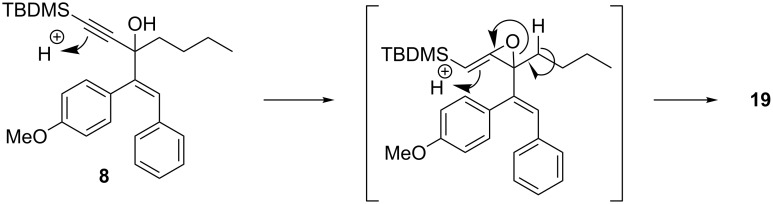
Proposed mechanism for the formation of enone **19**.

Remarkably, removal of the trimethoxyphenyl group suppressed the formation of cyclopentanone but promoted the formation of furan **18** (54% yield, entry 4, Table 1). Interestingly, substitution of the carbinol part by an *n*-butyl substituent provided an alternate type of product: the acyclic enone was the sole product isolated from the reaction medium (47% yield, entry 5, Table 1). It could be envisioned that this compound results from the rearrangement of an allene oxide (Scheme 5). Interestingly the dipropargylic alcohol **9** afforded the rearranged allylic ether **20** as the only isolated product (entry 6, Table 1).

In order to improve the yields of these reactions and gain insight in the reaction mechanisms, we explored different reaction conditions starting from alcohol **21** (Table 2). 1,2-Dichloroethane and dichloromethane (entries 1 and 2, Table 2) gave better yields (27–31% for **22** and 10–16% for **23**) compared to THF (entry 3, Table 2). In an effort to understand the role of Re_2_O_7_, the catalyst was changed to ReO_4_SiPh_3_ (entry 5, Table 2) and ReO_4_H (entry 6, Table 2). In both cases, the results were similar to those obtained with Re_2_O_7_. Removal of methanol from the medium suppressed the formation of **22** (entry 7, Table 2). In this case only furan **23** and rearranged alcohol **25** could be isolated as traces (5%).

**Table 2:** Optimization of the acid-catalyzed rearrangements of enynol **2**.

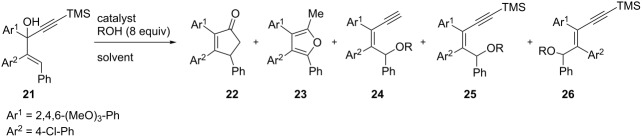									

Entry	R	Catalyst	Solvent	*T* (°C)	**22** (%)^a^	**23** (%)^a^	**24** (%)^a^	**25** (%)^a^	**26** (%)^a^

1	CH_3_	Re_2_O_7_	DCE	45	31	16	–	–	–
2	CH_3_	Re_2_O_7_	CH_2_Cl_2_	45	27	10	–	–	–
3	CH_3_	Re_2_O_7_	THF	45	11	9	–	12	–
4	CH_3_	Re_2_O_7_	THF	rt	12	–	–	4	–
5	CH_3_	ReO_4_SiPh_3_	DCE	45	28	17	6	–	–
6	CH_3_	ReO_4_H	DCE	45	30	17	–	–	–
7	–	ReO_4_H	DCE	45	–	5	–	5^b^	–
8	CH_3_	F_3_CSO_3_H	DCE	45	36	12	–	–	–
9	CH_3_	AcOH	DCE	45	–	–	–	51	47
10^c^	–	F_3_CSO_3_H	DCE	45	–	–	–	–	–
11^c^	CF_3_CH_3_	F_3_CSO_3_H	DCE	45	–	–	–	–	–
11	CH_3_	F_3_CSO_3_H	CH_3_NO_2_	45	30	–	–	–	–
12^c^	CH_3_	F_3_CSO_3_H	CH_3_NO_2_	rt	–	–	–	–	–

^a^Isolated yields. ^b^An allylic alcohol is generated (**25**, R = H). ^c^Degradation of the reaction mixture.

To confirm that the reaction is catalyzed by an acid, we changed its nature and tested two Brønsted acids. With F_3_CSO_3_H (entry 8, Table 2), we obtained comparable yields with 36% of cyclopentenone **22** and 12% of furan **23**. When using a weaker acid such as acetic acid (entry 9, Table 2) only the two acyclic ethers **25** and **26** were isolated. This observation strongly suggests that perrhenic acid is the active reagent generated from Re_2_O_7_ by methanolysis or hydrolysis with traces of water [12].

When removing methanol or replacing it with trifluoroethanol, the reaction mixture degraded in presence of triflic acid (entries 10 and 11, Table 2). Thus, the presence of methanol seems necessary for the cyclization to occur, perhaps by stabilizing an intermediate. Replacement of dichloroethane by nitromethane slightly decreased the yield of **22**, and suppressed the formation of furan **23**.

**Scheme 6:**
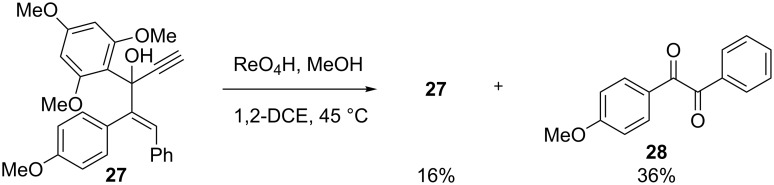
Rearrangement of unprotected propargylic carbinol **27**.

To determine whether the protection of the alkyne group was necessary for a rearrangement to take place, the reaction was carried out on compound **27**. In the presence of perrhenic acid, 16% of the starting material was recovered along with 36% of diketone **28** (Scheme 6). No cyclization product was isolated. This observation strengthened the hypothesis that the silyl group stabilizes the carbocation intermediate which seems necessary for the cyclization step.

## Conclusion

The discovery of new reaction manifolds often provides a good opportunity to discover novel reactivity in related systems. In this article, we demonstrate the delicate balance among several mechanistic pathways by a minor change of substrate in acid-catalyzed rearrangements of 1-styrylpropargyl alcohols. Indeed, these compounds may generate a furan (**18**), an enone (**19**), an allylic ether or even a naphthalenone (**17**). The formation of the latter is quite intriguing because it suggests an unprecedented cascade of reactions.

## Supporting Information

File 1 Experimental procedures for the synthesis of compounds **7**–**9**, **13**, **14** and **17**–**28**.
