# Supplementary material for: Novel carbocationic rearrangements of 1-styrylpropargyl alcohols
Source: Beilstein J Org Chem. 2015 Jun 15;11:1017–22. doi: 10.3762/bjoc.11.114 (PMC4505300; doi:10.3762/bjoc.11.114)
Supplement: File 1 — Experimental procedures for the synthesis of compounds 7–9, 13, 14 and 17–28. [file Beilstein_J_Org_Chem-11-1017-s001.pdf]

Supporting Information

for

Novel carbocationic rearrangements of 1-styrylpropargyl alcohols

Christine Basmadjian, Fan Zhang, and Laurent Désaubry*

Address: [1]Laboratory of Therapeutic Innovation (UMR 7200), University of Strasbourg - CNRS, Faculty of Pharmacy, 67401 Illkirch, France

Email: Laurent Désaubry - desaubry@unistra.fr

*Corresponding author

Experimental procedures for the synthesis of compounds **7–9**, **13**, **14** and **17–28**

NOE correlation between H^2 (δ 4.91 ppm) and H^3 (δ 7.18 ppm) and HMBC correlations between C^1 (δ 206.7 ppm) and H^1 (δ 1.65 ppm), and between H^2 (δ 4.91 ppm) and C^2 (δ 1.65 ppm).

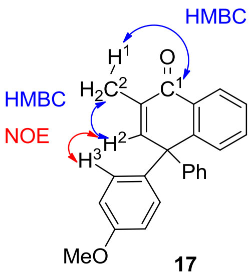

17

Figure S1: HMBC and NOESY correlation of methylnaphatalenone **17**.

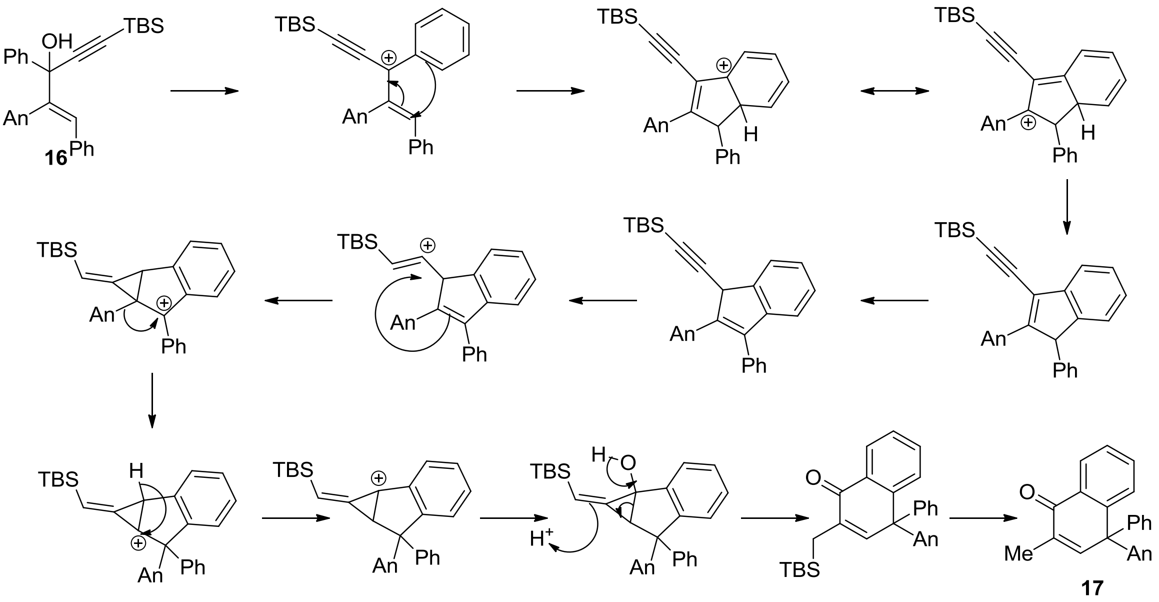

Scheme S1: Putative mechanism for the formation of methylnaphtalenone **17**. An = *p*-anisyl

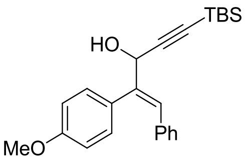

7

(*E*)-5-(*tert*-Butyldimethylsilyl)-2-(4-methoxyphenyl)-1-phenyl-pent-1-en-4-yn-3-ol (7). $NaBH_4$ (36.3 mg, 0.96 mmol) and $CeCl_3{\cdot}2H_2O$ (209 mg, 0.56 mmol) were added to a solution of ketone **6** (300 mg, 0.8 mmol) in anhydrous MeOH (5 mL) at 0 °C. After 2 h at 0 °C, the reaction mixture was quenched with water and diluted in Et_2O. The organic layer was separated, washed with brine, dried over $MgSO_4$, filtered and concentrated under reduced pressure to afford alcohol **7** with 76% yield (230 mg): ^{1}H NMR ($CDCl_3$, 400 MHz) δ 0.11 (s, 3H), 0.12 (s, 3H), 0.92 (s, 9H), 2.01 (d, 1H, J = 6.8 Hz), 3.82 (s, 3H), 5.17 (d, 1H, J = 6.8 Hz), 6.57–6.89 (m, 3H), 6.99–7.01 (m, 2H), 7.11–7.13 (m, 3H), 7.23 (d, 2H, J = 8.8 Hz) ppm; ^{13}C NMR ($CDCl_3$, 400 MHz) δ -4.5 (2C), 18.0, 26.2 (3C), 45.4, 68.6, 91.9, 111.3, 112.2, 114.3 (2C), 127.3 (2C), 128.1 (2C), 128.1, 129.5 (2C), 130.9 (2C), 131.0, 132.9 ppm; HR-MS calculated for $C_{24}H_{30}O_2Si$: 378.2015 , found: 401.1914 $(M+Na)^+$.

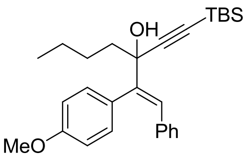

8

(*E*)-3-((*tert*-Butyldimethylsilyl)ethynyl)-2-(4-methoxyphenyl)-1-phenylhept-1-en-3-ol (8). At −78 °C, *n*-BuLi (1.6 M, 750 µL, 1.2 mmol) was added dropwise to ketone **6** (300 mg, 0.8 mmol) in anhydrous THF (5 mL) under an argon atmosphere. The resulting mixture was stirred at 0 °C for 3 h, then quenched with a saturated solution of $KHSO_4$. After extraction with Et_2O, the organic layer was dried over $MgSO_4$, filtered and concentrated. The crude product was purified on a silica column to give 166 mg (48%) of product **8:** ^{1}H NMR (CDCl$_3$, 400 MHz) δ 0.15 (s, 3H), 0.16 (s, 3H), 0.86–0.90 (m, 4H), 0.98 (s, 9H), 1.21–1.32 (m, 3H), 1.69 – 1.73 (m, 2H), 2.15 (s, 1H), 3.83 (s, 3H), 6.86–6.90 (m, 4H), 7.08–7.10 (m, 3H), 7.17 (d, 2H, 8.7 Hz), 7.19 (s, 1H) ppm; ^{13} C NMR (CDCl$_3$, 400 MHz) δ -4.5 (2C), 14.2, 16.8, 22.8, 26.3 (3C), 26.9, 40.6, 47.2, 55.3, 108.3, 114.0 (2C), 125.2, 127.0, 128.0 (2C), 128.6, 129.6 (2C), 131.7 (2C), 142.9, 159.2 ppm; HR-MS calculated for $C_{28}H_{38}O_2Si$: 434.2641, found: 457.2537 $(M+Na)^+$.

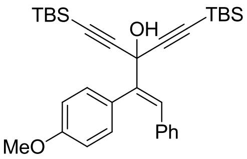

9

(*E*)-5-(*tert*-Butyldimethylsilyl)-3-((*tert*-butyldimethylsilyl)-ethynyl)-2-(4-methoxyphenyl)-1-phenylpent-1-en-4-yn-3-ol (9). *tert*-Butyldimethylsilylacetylene (180 µL, 0.95 mmol) was dissolved in anhydrous THF (2.5 mL) under an argon atmosphere. At −78 °C, *n*-BuLi (1.6 M, 630 µL, 1 mmol) was added dropwise and the mixture was stirred at 0 °C for 1 h. The obtained lithiated product was added dropwise at −78 °C to a solution of compound **5** (290 mg, 0.83 mmol) in anhydrous THF (2.5 mL). The obtained mixture was stirred at 0 °C for 3 h then the reaction was quenched with a saturated solution of $KHSO_4$. After extraction with Et_2O, the organic layer was dried over $MgSO_4$, filtered and concentrated. The crude product was purified on a silica column to give 128 mg (30%) of product **9**: ^{1}H NMR (CDCl$_3$, 400 MHz) δ 0.13 (s, 12H), 0.93 (s, 18H), 2.65 (s, 1H), 3.83 (s, 3H), 6.86 (d, 2H, J = 8.9 Hz), 6.93–7.12 (m, 5H), 7.29 (d, 2 H, J = 8.9 Hz), 7.34 (s, 1H) ppm; ^{13} C NMR (CDCl$_3$, 400 MHz) δ -4.7 (4C), 16.9 (2C), 26.2 (6C), 55.4, 67.9, 89.7, 104.7, 113.9 (2C), 127.4, 128.1 (2C), 128.5, 129.2, 129.7 (2C), 132.2 (2C), 136.2, 139.9, 159.5 ppm; HR-MS calculated for $C_{32}H_{44}O_2Si$: 516.2880, found: 529.2782 $(M+Na)^+$.

General procedure for the Re_2O_7-catalyzed rearrangements

Re_2O_7 (0.015 equiv) and MeOH (7.7 equiv) were dissolved in 1,2-DCE under an argon atmosphere, and the resulting mixture was stirred at 45 °C for 15 min. At 45 °C, a solution of propargylic alcohol (1 equiv) was added and the mixture was stirred at the same temperature overnight. The reaction was

then filtered on celite and concentrated to dryness. The obtained crude product was purified on silica gel.

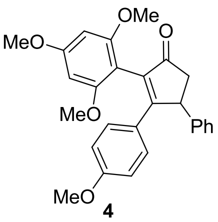

4

+

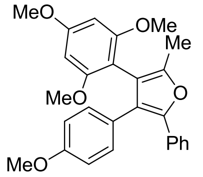

13

3-(4-Methoxyphenyl)-4-phenyl-2-(2,4,6-trimethoxyphenyl)cyclopent-2-en-1-one (4) and 3-(4-methoxyphenyl)-5-methyl-2-phenyl-4-(2,4,6-trimethoxyphenyl)furan (13). Compounds **4** and **13** were obtained simultaneously following the general procedure starting from propargylic alcohol **7** (9.3 g, 0.019 mol) in 1,2-DCE (60 mL) with Re_2O_7 (134 mg, 0.278 mmol) and MeOH (5 mL, 0.146 mol). Cyclopentenone **4** was obtained with 32% (2.46 g) along with methylfuran **13** (1.43 g, 18%). The NMR data for **4** were the same as those published in *Tetrahedron Lett.* **2015**, *56*, 727-730. HR-MS calculated for $C_{27}H_{26}O_5$: 430.1780, found: 431.1863 $(M+H)^+$.

Methylfuran **13**: ^{1}H NMR ($CDCl_3$, 400 MHz) δ 2.23 (s, 3H), 3.53 (s, 6H), 3.77 (s, 3H), 3.81 (s, 3H), 6.08 (s, 2H), 6.74 (d, 2H, *J* = 8.8 Hz), 7.03 (d, 2H, *J* = 8.8 Hz), 7.13 (t, 1H, *J* = 7.3 Hz), 7.21 (t, 2H, *J* = 7.8 Hz), 7.47 (d, 2H, *J* = 7.3 Hz) ppm; ^{13}C NMR ($CDCl_3$, 400 MHz) δ 11.9, 54.2, 54.4, 54.7 (2C), 89.8 (2C), 102.3, 112.4 (2C), 114.7, 123.3, 124.6 (2C), 125.9, 126.6, 127.2 (2C), 129.4 (2C), 131.0, 145.4, 148.3, 157.2, 158.1, 160.0 ppm; HR-MS calculated for $C_{27}H_{26}O_5$: 430.1780, found: 431.1854 $(M+H)^+$.

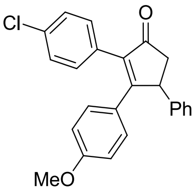

14

2-(4-Chlorophenyl)-3-(4-methoxyphenyl)-4-phenylcyclopent-2-en-1-one (14). Compound **14** was obtained following the general procedure starting from propargylic alcohol **15** (100 mg, 0.22 mmol) in 1,2-DCE (2 mL) with Re_2O_7 (1.45 mg, 0.003 mmol) and MeOH (65 µL, 1.58 mmol). Product **14** was obtained with 20% yield (15 mg). The NMR data for **14** were the same as those published in *Tetrahedron Lett.* **2015**, *56*, 727-730. HR-MS calculated for $C_{24}H_{19}ClO_2$: 374.1074, found: 375.1151 $(M+H)^+$.

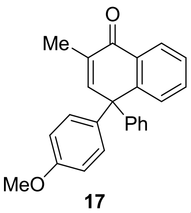

17 **4-(4-Methoxyphenyl)-2-methyl-4-phenylnaphthalen-1(4*H*)-one (17).** Compound **17** was obtained following the general procedure starting from propargylic alcohol **16** (100 mg, 0.22 mmol) in 1,2-DCE (2 mL) with Re_2O_7 (1.6 mg, 0.0033 mmol) and MeOH (70 µL, 1.77 mmol). Product **17** was obtained with 35% yield (26 mg). ^{1}H NMR ($CDCl_3$, 400 MHz) δ 1.65 (s, 3H), 3.77 (s, 3H), 4.91 (s, 1H), 6.74 (d, 2 H, *J* = 9.0 Hz), 7.18 (d, 2 H, *J* = 9.0 Hz), 7.23–7.49 (m, 9H) ppm; ^{13}C NMR ($CDCl_3$, 400 MHz) δ 24.3, 55.3, 67.2, 114.1 (2C), 120.8, 123.7, 126.1, 127.3, 128.0, 128.2, 129.2 (2C), 129.3 (2C), 130.0 (2C), 135.5, 139.6, 139.9, 141.6, 147.2, 159.2, 206.7 ppm; HR-MS calculated for $C_{24}H_{20}O_2$: 340.1463, found: 341.1536 $(M+H)^+$.

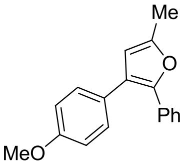

18 **3-(4-Methoxyphenyl)-5-methyl-2-phenylfuran (18).** Compound **18** was obtained following the general procedure starting from propargylic alcohol **7** (100 mg, 0.26 mmol) in 1,2-DCE (2 mL) with Re_2O_7 (1.92 mg, 0.004 mmol) and MeOH (80 µL, 2 mmol). Product **18** was obtained with 54% yield (37 mg). ^{1}H NMR ($CDCl_3$, 400 MHz) δ 2.38 (s, 3H), 3.83 (s, 3H), 6.12 (s, 1H), 6.88 (d, 2 H, *J* = 8.8 Hz), 7.17–7.51 (m, 5 H), 7.30 (d, 2 H, *J* = 8.8 Hz) ppm; ^{13}C NMR ($CDCl_3$, 400 MHz) δ 13.7, 55.3, 110.3, 114.1 (2C), 122.2, 125.8 (2C), 126.9, 127.1, 128.4 (2C), 129.8 (2C), 131.7, 146.5, 151.2, 158.8 ppm; HR-MS calculated for $C_{18}H_{16}O_2$: 264.1150, found: 265.1226 $(M+H)^+$.

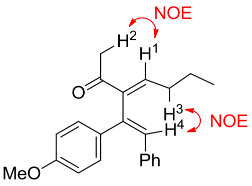

19 **(*E*)–3-((*E*)-1-(4-Methoxyphenyl)-2-phenylvinyl)hept-3-en-2-one (19).** Compound **19** was obtained following the general procedure starting from propargylic alcohol **8** (100 mg, 0.23 mmol) in 1,2-DCE (2 mL) with Re_2O_7 (1.67 mg, 0.0035 mmol) and MeOH (70 µL, 1.77 mmol). Product **19** was obtained with 47% yield (35 mg). ^{1}H NMR ($CDCl_3$, 400 MHz) δ 0.96 (t, 3H, *J* = 7.3 Hz), 1.54 (sex., 2H, *J* = 7.3 Hz), 2.16 (s, 3H), 2.38 (quad., 2H, *J* = 7.4 Hz), 3.78 (s, 3H), 6.42 (s, 1H), 6.75 (d, 2H, *J* = 8.9 Hz), 6.83 (t, 1H, *J* = 7.5 Hz), 7.09 (d, 2H, *J* = 8.9 Hz), 7.14–7.19 (m, 5H) ppm; ^{13}C NMR ($CDCl_3$, 400 MHz) δ 14.2, 22.5, 27.7, 31.8, 55.3, 113.9 (2C), 127.0, 128.2 (2C), 129.4 (2C), 130.6 (2C), 131.1, 136.7, 137.1, 143.6, 145.3, 159.*1*, 199.2 ppm; HR-MS calculated for $C_{22}H_{24}O_2$: 320.1776, found: 321.1858 $(M+H)^+$. The structure of ketone **19** was confirmed by NMR 2D

experiment. NOESY correlations were observed between H^1 (δ 6.83 ppm) and H^2 (δ 2.16 ppm), and also between H^3 (δ 2.38 ppm) and H^4 (δ 6.42 ppm).

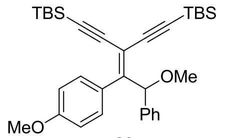

20 **(3-(2-Methoxy-1-(4-methoxyphenyl)-2-phenylethylidene)penta-1,4-diyne-1,5-diyl)bis(*tert*-butyldimethylsilane) (20).** Compound **20** was obtained following the general procedure starting from propargylic alcohol **9** (100 mg, 0.19 mmol) in 1,2-DCE (2 mL) with Re_2O_7 (1.4 mg, 0.0029 mmol) and MeOH (60 µL, 1.46 mmol). Product **20** was obtained with 31% yield (31 mg**).** ^{1}H NMR ($CDCl_3$, 400 MHz) δ -0.01 (s, 3H), 0.00 (s, 3H), 0.20 (s, 6H), 0.78 (s, 9H), 0.99 (s, 9H), 3.47 (s, 3H), 3.75 (s, 3H), 6.00 (s, 1H), 6.69 (d, 2H, *J* = 8.8 Hz), 7.04 (d, 2H, *J* = 8.8 Hz), 7.19 – 7.31 (m, 5H) ppm; ^{13} C NMR ($CDCl_3$, 400 MHz) δ -4.6 (2C), -4.8 (2C), 16.8, 17.0, 26.1 (3C), 26.3 (3C), 55.3, 57.1, 83.0, 96.4, 98.3, 102.2, 102.4, 106.4, 112.9 (2C), 126.2 (2C), 127.3, 128.2 (2C), 128.4, 130.9 (2C), 140.0, 157.5, 159.5 ppm; HR-MS calculated for $C_{33}H_{46}O_2Si_2$: 530.3036, found: 553.2922 $(M+Na)^+$.

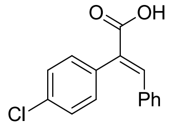

21-1 **(*E*)-2-(4-Chlorophenyl)-3-phenylacrylic acid (21-1).** Compound **21-1** was obtained following the procedure in reference [4] starting from *p*-chlorophenylacetic acid (28 g, 0.163 mol) in acetic anhydride (62 mL) with benzaldehyde (18.4 mL, 0.182 mol) and triethylamine (22.3 mL, 0.2 mol). Product **21-1** was obtained quantitatively (50 g). ^{1}H NMR ($CDCl_3$, 400 MHz) δ 7.088 (d, 2H, *J* = 7.2 Hz), 7.17–7.27 (m, 5H), 7.36 (d, 2H, *J* = 8.5 Hz), 7.98 (s, 1H) ppm; ^{13}C NMR ($CDCl_3$, 400 MHz) δ 128.6 (2C), 129.2 (2C), 129.9, 130.5, 130.9 (2C), 131.5 (2C), 133.8, 134.1, 134.3, 142.3, 172.7 ppm; BR-MS calculated for $C_{15}H_{11}ClO_2$: 258.04, found: 258.0 $(M+H)^+$.

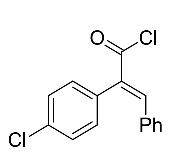

21-2

(*E*)-2-(4-chlorophenyl)-3-phenylacryloyl chloride (21-2). Compound **21-2** was obtained following the procedure in reference [4] starting from carboxylic acid **21-1** (9.9 g, 0.039 mol) in CH_2Cl_2 (50 mL) with DMF (70 µL, 1 mmol) and oxalyl chloride (3.8 ml, 0.045 mol). Product **21-2** was obtained quantitatively (10 g). ^{1}H NMR ($CDCl_3$, 400 MHz) δ 7.72 (d, 2H, *J* = 7.5 Hz), 7.18 (d, 2H, *J* = 8.5 Hz),

7.25 (t, 2H, J = 7.9 Hz), 7.32–7.36 (m, 1H), 7.41 (d, 2H, J = 8.4 Hz) ppm; ^{13}C NMR ($CDCl_3$, 400 MHz) δ 128.3, 128.5 (2C), 129.1 (2C), 129.9, 130.4, 130.9 (2C), 131.5 (2C), 133.7, 137.1, 143.3, 172.6 ppm.

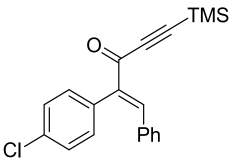

21-3

(*E*)-2-(4- Chlorophenyl)-1-phenyl-5-(trimethylsilyl)pent-1-en-4-yn-3-one (21-3). Compound **21-3** was obtained following the procedure in reference [4] starting from acyl chloride **21-2** (10 g, 0.037 mol) in 1,4-dioxane (280 mL) with trimethylsilylacetylene (5.27 mL, 0.037 mol), $Pd(PPh_3)_2Cl_2$ (512 mg, 0.73 mmol), CuI (282 mg, 1.48 mmol) and Et_3N (15.5 mL, 0.111 mol). Product **21-3** was obtained with 80% yield (10 g). ^{1}H NMR ($CDCl_3$, 400 MHz) δ 0.287 (s, 9H), 7.09–7.13 (m, 4H), 7.22–7.30 (m, 3H), 7.37 (d, 2H, J = 8.7 Hz), 8.13 (s, 1H) ppm; ^{13}C NMR ($CDCl_3$, 400MHz) δ -0.6 (3C), 100.7, 101.0, 128.7 (2C), 129.2 (2C), 130.3, 131.2 (2C), 131.5 (2C), 133.0, 134.1, 134.4, 140.3, 146.4, 178.7 ppm.

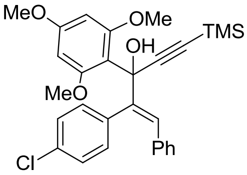

21

(*E*)-2-(4-Chlorophenyl)-1-phenyl-3-(2,4,6-trimethoxyphenyl)-5-(tri-methylsilyl)pent-1-en-4-yn-3-ol (21). Compound **21** was obtained following the procedure in reference [4] starting from ketone **21-3** (5 g, 0.015 mol) in THF (110 mL) with 2,4,6-trimethoxybenzene (7.4 g, 0.044 mol) and *sec*-BuLi (1.3 M, 33.8 mL, 0.044 mol). Product **21** was obtained with 46% yield (3.5 g). ^{1}H NMR ($CDCl_3$, 400 MHz) δ 0.20 (s, 9H), 3.65 (s, 6H), 3.79 (s, 3H), 6.07 (s, 2H), 6.57 (s, 1H), 6.88–6.90 (m, 2H), 6.97 (d, 2H, J = 8.4 Hz), 7.06–7.10 (m, 3H), 7.15 (d, 2H, J = 8.5 Hz), 7.21 (s, 1H) ppm; ^{13}C NMR ($CDCl_3$, 400 MHz) δ 0.1 (3C), 55.2, 56.1 (2C), 88.8, 92.6 (2C), 107.3, 110.7, 126.7, 127.2, 127.7 (2C), 127.8 (2C), 129.3 (2C), 132.1 (2C), 132.8, 136.6, 136.8, 142.7, 158.7, 160.5 ppm; HR-MS calculated for $C_{29}H_{31}ClO_4Si$: 506.1651, found: 529.1573 $(M+Na)^+$.

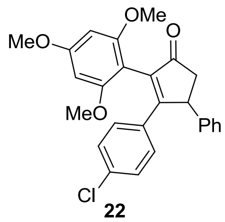

22

+

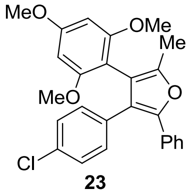

23 **3-(4-Chlorophenyl)-4-phenyl-2-(2,4,6-trimethoxyphenyl)cyclopent-2-en-1-one (22) and 3-(4-chlorophenyl)-5-methyl-2-phenyl-4-(2,4,6-trimethoxyphenyl)furan (23).** Compounds **22** and **23** were obtained following the general procedure starting from propargylic **21** (150 mg, 0.296 mmol) in 1,2-DCE (2 mL) with Re_2O_7 (2.1 mg, 0.0044 mmol) and MeOH (92 µL, 2.28 mmol). Cyclopentenone **22** (40 mg, 31%) was obtained along with methylfurane **23** (21 mg, 16%). Cyclopentenone **22**: ^{1}H NMR ($CDCl_3$, 400 MHz) δ 2.45 (dd, 1H , *J* = 1.9 and 18.4 Hz), 3.19 (dd, 1H, *J* = 7.4 and 18.6 Hz), 3.53 (s, 3H), 3.77 (s, 3H), 3.84 (s, 3H), 4.60 (dd, 1H, *J* = 2.0 and 7.4 Hz), 6.12 (d, 1H, *J* = 2.1 Hz), 6.22 (d, 1H, *J* = 2.1 Hz), 7.07 (d, 2H, *J* = 8.8 Hz), 7.18–7.16 (m, 3H), 7.25–7.27 (m, 4 H) ppm; ^{13}C NMR ($CDCl_3$, 400 MHz) δ 45.9, 47.4, 55.5, 55.8, 56.1, 66.0, 91.4, 91.5, 127.0, 127.5 (2C), 128.3 (2C), 129.1 (2C), 129.3 (2C), 134.3, 135.0, 136.5, 143.3, 158.5, 159.1, 162.1, 168.3, 207.1 ppm; HR-MS calculated for $C_{26}H_{23}ClO_4$: 434.1285, found: 435.1359 $(M+H)^+$. Methylfurane **23**: ^{1}H NMR ($CDCl_3$, 400 MHz) δ 2.24 (s, 3H), 3.53 (s, 6H), 3.82 (s, 3H), 6.09 (s, 2H), 7.08 (d, 2H, *J* = 8.7 Hz), 7.15–7.18 (m, 3H), 7.22–7.24 (m, 2H), 7.45 (d, 2H, *J* = 7.0 Hz) ppm; ^{13}C NMR ($CDCl_3$, 400 MHz) δ 14.4, 54.4, 54.6 (2C), 65.0, 89.8 (2C), 101.7, 114.4, 122.3, 124.9 (2C), 125.8, 127.2 (2C), 127.3 (2C), 129.6 (2C), 130.6, 131.2, 132.9, 145.8, 148.7, 158.0, 160.2 ppm; HR-MS calculated $C_{26}H_{23}ClO_4$: 434.1285, found: 435.1363 $(M+H)^+$.

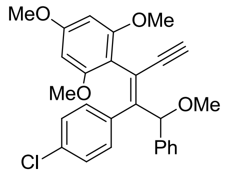

24 **(*E*)-2-(4-(4-Chlorophenyl)-5-methoxy-5-phenylpent-3-en-1-yn-3-yl)-1,3,5-trimethoxybenzene (24)** Compound **24** was obtained following general procedure starting from compound **21** (100 mg, 0.197 mmol) in 1,2-DCE (2 mL) with ReO_4SiPh_3 (1.5 mg, 0.003 mmol) and MeOH (61 µL, 1.52 mmol). ^{1}H NMR ($CDCl_3$, 400 MHz) δ 3.23 (s, 1H), 3.62 (s, 3H), 3.64 (s, 3H), 3.72 (s, 3H), 3.74 (s, 3H), 5.91 (d, 1H, *J* = 2.1 Hz), 5.95 (d, 1H, *J* = 2.1 Hz), 6.14 (s, 1H), 6.62 (d, 2H, *J* = 8.5 Hz), 6.87 (d, 2H, 8.5 Hz), 7.21 – 7.24 (m, 1H), 7.26 – 7.28 (m, 2H), 7.43 (d, 2H, *J* = 8.0 Hz) ppm.

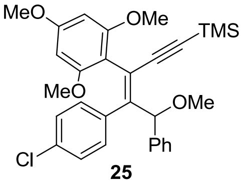

25

+

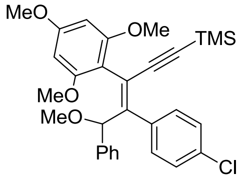

26

(*Z*)-(4-(4-Chlorophenyl)-5-methoxy-5-phenyl-3-(2,4,6-trimethoxyphenyl)pent-3-en-1-yn-1-yl)trimethylsilane (25) and (*E*)-(4-(4-chlorophenyl)-5-methoxy-5-phenyl-3-(2,4,6-trimethoxyphenyl)-pent-3-en-1-yn-1-yl)trimethylsilane (26). MeOH (62 µL, 1.52 mmol) and AcOH (3 µL, 0.049 mmol) were added to a solution of propargylic alcohol **21** in anhydrous 1,2-DCE (2 mL) under an argon atmosphere. The reaction mixture was stirred overnight at 45 °C. Concentration under reduced pressure and purification of the crude over silica gel afforded compounds **25** (53 mg, 51%) and **26** (48 mg, 47%). Compound **25**: ^{1}H NMR ($CDCl_3$, 400 MHz) δ 0.19 (s, 9H), 6.61 (s, 6H), 3.69 (s, 3H), 3.72 (s, 3H), 5.91 (d, 1H, *J* = 2.1 Hz), 5.93 (d, 1H, *J* = 2.1 Hz), 6.18 (s, 1H), 6.62 (d, 2H, *J* = 8.5 Hz), 6.86 (d, 2H, *J* = 8.5 Hz), 7.20 (t, 1H, *J* = 7.2 Hz), 7.27 (t, 2H, *J* = 7.2 Hz), 7.40 (d, 2H, *J* = 7.9 Hz) ppm; ^{13}C NMR ($CDCl_3$, 400 MHz) δ 0.1 (3C), 55.2, 55.7, 55.7, 56.6, 83.1, 90.8 (2C), 97.8, 104.3, 109.3, 118.2, 126.7 (2C), 126.7 (2C), 126.1, 127.8 (2C), 130.1 (2C), 132.4, 135.9, 140.5, 149.8, 157.9, 158.0, 161.0 ppm; HR-MS calculated for $C_{30}H_{33}ClO_4Si$: 520.1837, found: 521.1907 $(M+H)^+$. Compound **26**: ^{1}H NMR ($CDCl_3$, 400 MHz) δ -0.05 (s, 9H), 3.37 (s, 3H), 3.82 (s, 3H), 3.85 (s, 3H), 3.86 (s, 3H), 4.99 (s, 1H), 6.20 (d, 1H, *J* = 2.3 Hz), 6.21 (d, 1H, *J* = 2.1 Hz), 7.14–7.21 (m, 9H) ppm; ^{13}C NMR ($CDCl_3$, 400 MHz) δ -0.1 (3C), 55.5, 55.7, 55.9, 56.9, 81.8, 91.1, 91.2, 96.8, 105.4, 107.9, 118.5, 126.8 (2C), 126.9, 127.3 (2C), 127.7 (2C), 131.8 (2C), 132.9, 137.0, 140.4, 149.8, 158.2, 158.8, 161.5 ppm; HR-MS calculated for $C_{30}H_{33}ClO_4Si$: 520.1837, found: 521.736 $(M+H)^+$.

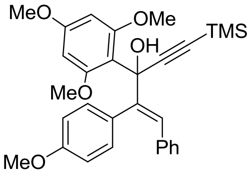

10

+

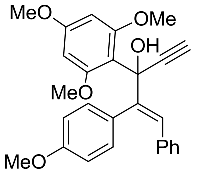

27

(*E*)-2-(4-Methoxyphenyl)-1-phenyl-3-(2,4,6-trimethoxyphenyl)-5-(trimethylsilyl)pent-1-en-4-yn-3-ol (10) and (*E*)–2-(4-methoxyphenyl)-1-phenyl-3-(2,4,6-trimethoxyphenyl)pent-1-en-4-yn-3-ol (27). Compound **10** was obtained following the procedure in reference [4] with 71% yield (26.6 g) along with deprotected alkyne **27** (1.1g, 14%). Compound **10**: ^{1}H NMR ($CDCl_3$, 400 MHz) δ 0.21 (s, 9H), 3.63 (s, 6H), 3.77 (s, 3H), 3.78 (s, 3H), 6.07 (s, 2H), 6.50 (s, 1H), 6.51 (d, 2H, *J* = 8.7 Hz), 6.89–6.92 (m, 4H), 7.04 – 7.07 (m, 3H), 7.21 (s, 1H) ppm; ^{13}C NMR ($CDCl_3$, 400 MHz) δ 0.126 (3C), 55.1, 55.2, 56.2 (2C), 88.5, 92.7 (2C), 107.7, 113.0, 113.1 (2C), 114.1, 114.3, 126.4, 126.8, 127.7 (2C), 129.3 (2C), 130.1, 131.0, 131.7 (2C), 137.3, 143.5, 158.6, 158.8 ppm; HR-MS calculated for $C_{30}H_{34}O_5Si$: 502.2176, found: 525.2077 $(M+Na)^+$. Compound **27**: ^{1}H NMR ($CDCl_3$, 400 MHz) δ 3.65 (s, 6H), 3.75 (s, 3H), 3.77 (s, 3H), 5.83 (s, 1H), 6.05 (s, 2H), 6.75 (d, 2H, *J* = 8.8 Hz), 7.18–7.29 (m, 6H), 7.75 (d, 2H, *J* = 8.8 Hz) ppm; ^{13}C NMR ($CDCl_3$, 400 MHz) δ 55.4 (2C), 55.9 (2C), 91.3 (2C), 111.3, 113.2 (2C), 126.4, 128.0 (2C), 129.8 (2C), 130.1 (2C), 130.6, 139.5, 158.1 (2C), 160.7, 162.4, 198.2 ppm.

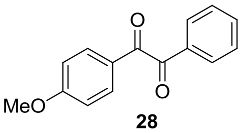

28

1-(4-Methoxyphenyl)-2-phenylethane-1,2-dione (28). MeOH (109 µL, 2.68 mmol) and ReO_4H/H_2O (50/50, v/v, 2.6 µL) were added to a solution of compound **27** in anhydrous 1,2-DCE (2 mL) under an argon atmosphere. The reaction mixture was stirred 48 h at 45 °C, concentrated and purified over silica gel to give compound **28** (30 mg, 36%). ^{1}H NMR ($CDCl_3$, 400 MHz) δ 3.89 (s, 3H), 6.98 (d, 2H, *J* = 8.9 Hz), 7.50 (t, 2H, *J* = 8.0 Hz), 7.65 (t, 1H, *J* = 7.4 Hz), 7.94 – 7.98 (m, 4 H) ppm; ^{13}C NMR ($CDCl_3$, 400 MHz) δ 55.8, 114.5 (2C), 126.3, 129.1 (2C), 130.1 (2C), 132.5 (2C), 133.4, 134.8, 165.1, 193.3, 195.0 ppm.
